# Precocious puberty in boys: current insights into etiology, genetic advances, and environmental factors

**DOI:** 10.3389/fendo.2026.1874027

**Published:** 2026-07-16

**Authors:** Maria Elisa Amodeo, Giulia Mirra, Annalisa Deodati, Stefano Cianfarani

**Affiliations:** 1 Endocrinology and Diabetes Unit, IRCCS “Bambino Gesù” Children’s Hospital, Rome, Italy; 2 Department of Systems Medicine, University of Rome Tor Vergata, Rome, Italy; 3 Department of Women's and Children's Health, Karolinska Institute, Stockholm, Sweden

**Keywords:** boys, brain lesions, endocrine disruptors, PFAS, precocious puberty

## Abstract

**Background:**

Central precocious puberty (CPP) in males results from the premature activation of the hypothalamic–pituitary–gonadal (HPG) axis, clinically diagnosed by a testicular volume >4 mL before the age of 9 years. Over the past two decades, a clear secular trend toward earlier pubertal onset has been reported. Historically, CPP in boys was strongly associated with intracranial lesions, observed in 40–50% of cases.

**Recent findings:**

Over the last decade, converging evidence has indicated a markedly lower prevalence of brain lesions in males with CPP, approximately 6–8%. Identified risk factors for intracranial lesions include neurological symptoms, pubertal onset before 8 years, and maternal age at menarche above 11 years. These data support a more selective, risk-based approach to neuroimaging and highlight the need to re-define a new consensus, recently published. Idiopathic CPP represents the majority of cases also in males, with increasing evidence supporting a strong genetic basis. Key mutations include gain-of-function variants in KISS1 and KISS1R, as well as loss-of-function mutations in MKRN3 and DLK1. Additional candidate genes—LIN28B, GABRA1, NPYR, TAC3, and TACR3—have been recently linked to pubertal regulation, although their precise mechanistic roles remain unclear. Beyond genetics, environmental exposures, particularly to endocrine-disrupting chemicals (EDCs), have been implicated in modulating pubertal timing.

**Conclusion:**

CPP in boys results from a multifactorial interplay between genetic predisposition and environmental influences. This review summarizes recent evidence regarding the prevalence of brain lesions, emerging genetic discoveries, and the role of endocrine disruptors, to promote a more personalized and precise diagnostic algorithm.

## Introduction

Central precocious puberty (CPP) in boys is defined as the premature activation of the hypothalamic–pituitary–gonadal (HPG) axis, clinically identified by testicular enlargement ≥4 mL before the age of 9 years (1). Although CPP is markedly less frequent in males than in females—affecting approximately 1 in 5,000–10,000 boys—it has historically been regarded as a high-risk condition because of its strong association with central nervous system organic conditions (2).

Previous literature reported pathological brain lesions in up to 40–75% of boys with CPP, leading to long-standing recommendations for routine neuroimaging in all male patients (3). However, these estimates were largely derived from small, heterogeneous cohorts, often including boys with peripheral precocious puberty, pre-existing neurological disorders, or prior oncological treatments. Over the last two decades, a profound epidemiological shift has emerged, characterized by an increasing incidence of idiopathic CPP and a parallel reduction of organic causes, potentially influenced by other factors such as endocrine disruptors (4, 5).

This review is focuses on new clinical, genetic, and environmental evidence in male CPP. Particular emphasis is placed on the updated prevalence of intracranial lesions, emerging risk stratification tools for neuroimaging, advances in the genetic architecture of idiopathic CPP, and the potential role of endocrine-disrupting chemicals (EDCs) and early-life factors in modulating pubertal timing.

## Secular trends in male CPP and the impact of COVID19 pandemic

CPP is approximately ten times more frequent in girls than in boys; nevertheless, recent epidemiological studies have demonstrated a consistent increase in male CPP diagnoses over time (6). In the United States, the prevalence rose from 6.2 per 100,000 boys in 1997 to 10.0 per 100,000 in 2010 mirroring trends observed in several European and Asian cohorts (7). This secular trend toward earlier pubertal onset appears to be independent of height acceleration and likely reflects a complex interplay between genetic susceptibility and environmental modulation.

The COVID-19 pandemic further accentuated this trend: several studies reported an abrupt rise in new CPP diagnoses during and after the lockdown period particularly in girls (8). Lifestyle changes—including reduced physical activity, increased screen exposure, altered sleep patterns, and psychosocial stress—have been hypothesized to contribute to earlier reactivation of the HPG axis, although causal relationships remain unproven (9–11).

Very few data have shown a clear link between COVID19 pandemic and the CPP incidence among boys. In a recent review including 26 studies from 11 countries, four studies reported no significant change in case numbers of CPP in boys, three studies observed a 2.8- to 3.4-fold increase, and one study found a 75% decrease in incidence (12). Twelve studies documented a rise in electronic device use and sedentary behavior, along with increased weight and body mass index Z-scores, more frequent sleep disturbances, and a younger age at pubertal onset.

In a recent Italian study conducted on 102 boys, from 2000 onward, new diagnoses of precocious puberty in boys progressively increased and 21 new cases occurred during the pandemic period (2020–2022), representing 20.6% of all diagnoses (13). Most of them were idiopathic and only one case was associated with a pathological MRI finding (hamartoma).

Subsequently, several region-specific prevalence studies conducted in Japan, Turkey, and Portugal further confirmed the year-by-year increase in the incidence of male central precocious puberty, with a particularly marked rise observed during the SARS-CoV-2 pandemic period (14–16).

## Evaluation of the risk for intracranial lesions in boys with CPP

Seminal studies from the 1980s and 1990s reported intracranial lesions in 70–90% of boys with precocious puberty (17). These cohorts frequently included patients with hypothalamic hamartomas, optic gliomas, pineal tumors, tuberculomas, and sequelae of CNS insults. Importantly, many studies did not distinguish between central and peripheral conditions of precocious puberty, included mixed cohorts (female and males), did not consider other risk factors and lacked stratification by age at onset or clinical presentation (**Table 1**).

**Table 1:** Relevant previous studies from 2000 to 2026 reporting the occurrence and the type of CNS lesions in a male cohort with precocious puberty across the world and potential confounding factors.

Author, year	Country	Cases	% organic	Type of CNS lesions	Confounders
De Sanctis, 2000 (42)	Italy	45	46%	13 intracranial lesions and 5 with intracranial anomaly. 6 hamartomas of the tuber cinereum, the others with: craniopharyngioma, 5 neurofibromatosis, post RT ependymoma, post-meningitis, CMV infection, post CT	RT, NF1 VT > 3 ml
Chemaitilly, 2001 (43)	France	26	73%	14 previously treated CNS lesions (surgery, RT, CT) 5 new lesions (3 hamartomas, 2 optic gliomas)	RT, CT, surgery M and F
Topor, 2018 (44)	USA	50	64%	10 neurofibromatosis type1, 4 optic glioma, 3 hypothalamic hamartoma, 3 other CNS tumor, 12 genetic syndromes/brain injury	NF1, Genetic Syndromes
Yoon, 2018 (45)	Korea	138	7%	6 pineal or arachnoid or Rathke Cysts, 3 pituitary hyperplasia, 1 thick pituitary stalk	Precocious and early puberty (children 9–10 years)
Vurallı, 2020 (46)	Turkey	120	21.7%	16 new lesions with 6 hamartomas, 4 arachnoid cysts, 2 gliomas, 1 craniopharyngioma, 1 germonima, 1 pinealoblastoma, 1 hemorragic adenoma. 10 (8.3%) with previous developmental anomaly of CNS (parenchymal injury, necrotic lesions and hydrocephalus)	Previous CNS anomalies
Wang, 2021 (47)	China	129	16.3%	1 not-secreting pineal germinoma 1 hypothalamic hamartoma 18 others (8 Rathke or arachnoid or pineal cysts,7 pituitary hyperplasia, 4 pituitary hypoplasia)	Precocious and early puberty (children 9–10 years)
Bajpai, 2022 (48)	India	26	55%	4 hamartomas, 2 craniopharingioma, 1 dysgerminoma. Other causes of neuro-genic CPP included neurotuberculosis, hydrocephalus, post radiotherapy, oloprosencehaly	Central and peripheral PP
Kendirci, 2022 (49)	Europe	9	55%	1 microadenoma 4 other findings (altered pituitary enhancement, demyelination area, corpus callosum dysgenesis, cavum septum pellucidum alteration)	Previous brain tumors
Hansen, 2023 (50)	Denmark	24	10.3%	Hamartomas and other lesions not specified	Mixed cohort M and F
Cassio, 2024 (5)	Italy	193	5.7%	5 Hypothalamic hamartoma 3 Optic glioma 1 Germinoma non-hCG secreting 1 Pilocytic astrocytoma 1 Craniopharyngioma	-
Amodeo, 2024 (13)	Italy	102	7.8%	4 Hypothalamic Hamartomas 2 Diencephalic Gangliogliomas 1 Diencephalic Low-Grade-Astrocytoma 1 Pineal Gland Germinoma	–

RT, Radiotherapy, CT, Chemiotherapy, NF1, Neurofibromatosis Type 1, M, Male, F, Female, CNS, Central Nervous System.

More recent, methodologically robust studies have consistently demonstrated a markedly lower prevalence of pathological brain lesions in boys with CPP, currently estimated at approximately 6–8% (5, 13). Large cohorts with careful exclusion of confounding neurological conditions show that the majority of male CPP cases are now idiopathic, with normal MRI findings or only mild, incidental CNS abnormalities (**Supplementary Table 2 - Supplementary Files**).

Age at pubertal onset has emerged as a critical determinant of risk. Pathological CNS lesions are predominantly identified in boys with very early onset CPP (before 6–8 years), whereas the likelihood of detecting a tumor in boys presenting after 8 years of age without neurological symptoms is exceedingly low. A recent review supporting the Endocrine Society Clinical Practice Guidelines reported a pathogenic lesion rate of 28.06% in boys aged <8 years. Specifically, hamartomas were identified in 12.95% of boys aged <8 years and in 0% of those aged 8–9 years. CNS Tumors were detected in 15.11% of boys aged <8 years and in 0% of those aged 8–9 years (51).

## Clinical predictors of organic central precocious puberty in boys

Across contemporary studies, several clinical and historical features consistently predict an increased risk of intracranial pathology (5, 13):

- pubertal onset before 8 years of age, in particular before 6 years;
- rapidly progressive pubertal development with accelerated growth velocity and bone age advancement;
- neurological signs or symptoms (headache, visual disturbances, vomiting, cranial nerve deficits);
- absence of a familial history of early or precocious puberty.

Conversely, a positive family history and early maternal age at menarche are strongly associated with idiopathic CPP, reinforcing the role of heritable factors in male pubertal timing.

**Figure 1:**
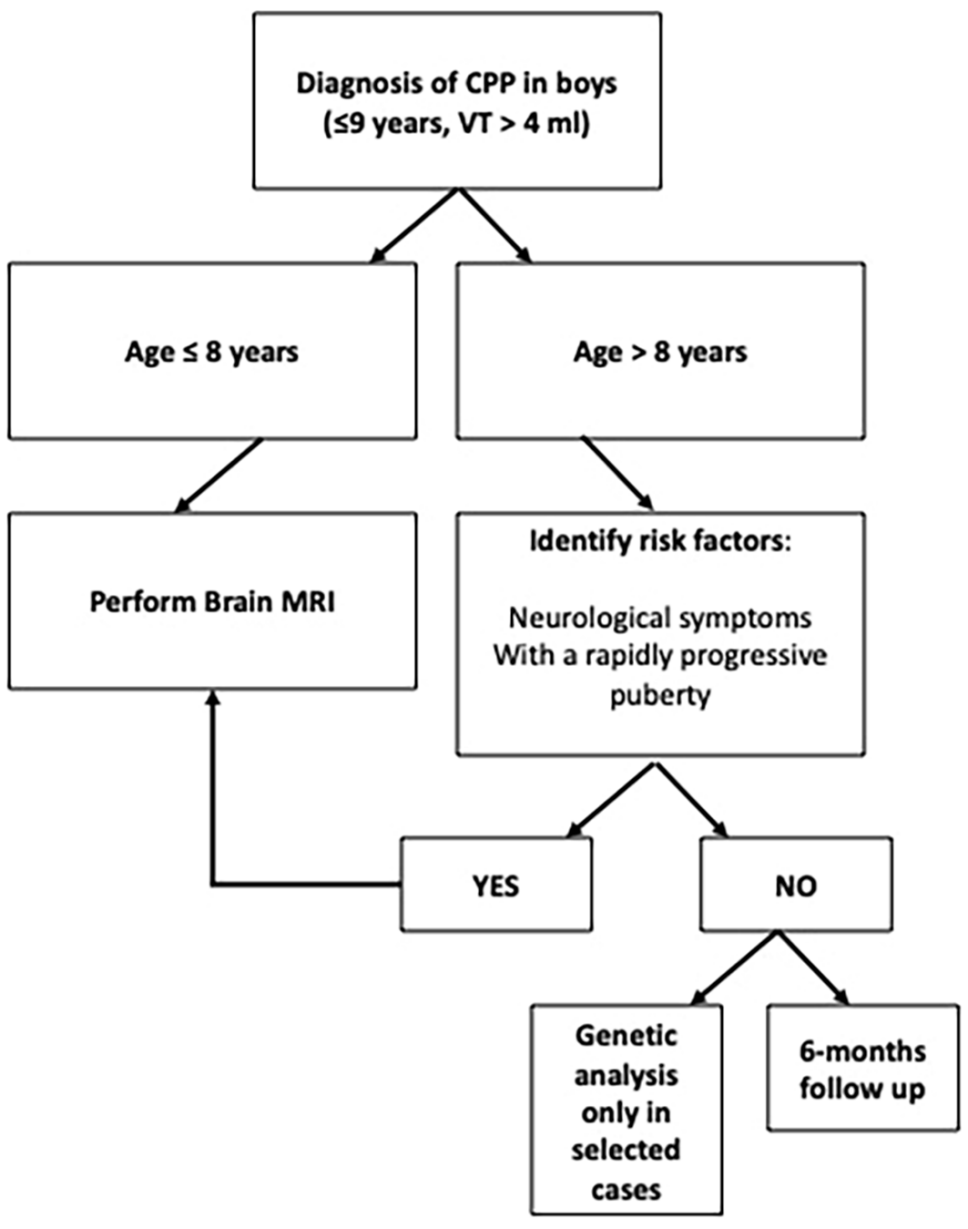
Diagnostic algorithm for precocious puberty in boys.

These observations argue against routine MRI screening and instead favor a stratified, risk-oriented imaging strategy in boys with CPP (**Figure 1**). According to the latest Endocrine Society Clinical Practice Guideline, brain MRI should not be routinely performed in boys aged 8–9 years in the absence of neurological signs or symptoms (52). Neuroimaging should be prioritized in younger patients and in those with rapid progression or neurological symptoms, while a more conservative strategy may be appropriate in older boys with slow progression and familial clustering. This approach reduces unnecessary sedation, costs (especially for low-income regions), and parental anxiety without compromising diagnostic safety.

**Figure 2:**
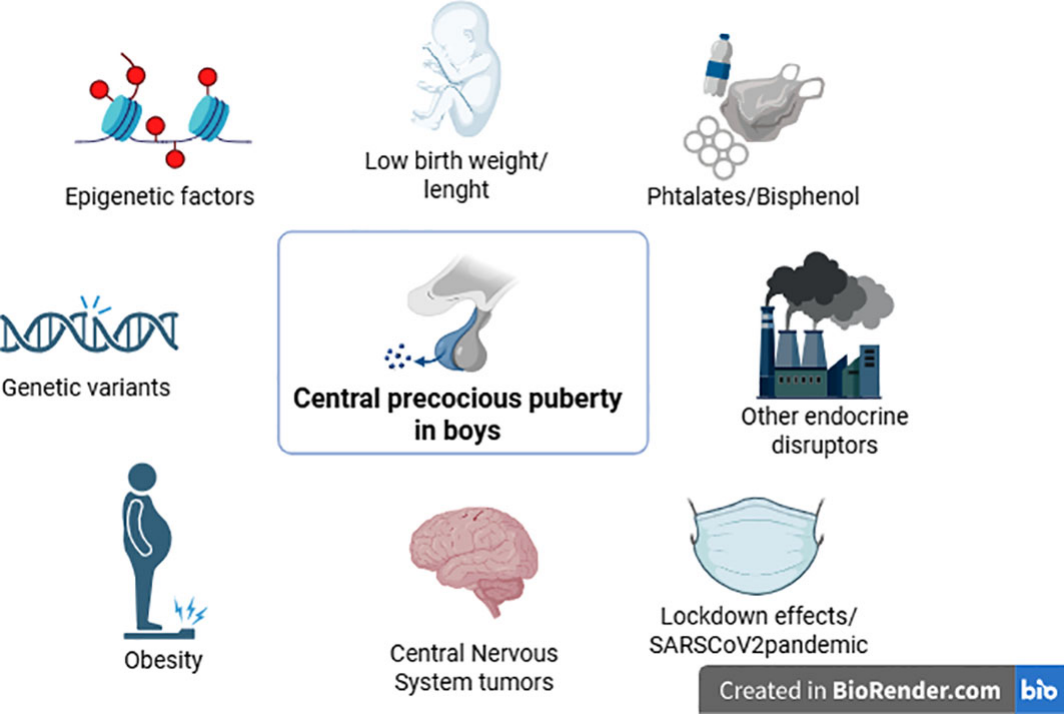
Causes of central precocious puberty in boys. Original illustration Created with BioRender.com.

The most recent studies reported in the literature described a male cohort of children with a more rigorous diagnosis of CPP, with no potentially confounding factors, accurately selected, specifically excluding the following confounding factors (5, 13):

- a known history of previous brain lesions;
- any treatments that may have influenced the onset of PP (radiotherapy, chemotherapy, intracranial procedures, sex hormones treatments);
- diseases/syndromes already known in literature to correlate with early puberty or CNS tumors (neurological diseases, epilepsy, syndromes such as neurofibromatosis, Williams Syndrome, CNS severe malformations);
- other endocrinological risk factors (CAH, Klinefelter Syndrome, McCune Albright Syndrome).

Probably for this reason, the percentage of pathological lesions at MRI is clearly lower than in other studies reported in the literature, making the diagnosis less suggestive of an underlying organic etiology.

Reviewing critically the studies reported in literature, the new proposed recommendation is to lower the age threshold for routine brain MRI in CPP to 8 years in boys, except those with rapidly progressing puberty, no positive family history for CPP and neurological symptoms of concern. This new indication will be verified in further multicenter studies with a larger cohort. Based on a critical review of the available evidence, a revised recommendation has been proposed to lower the age threshold for routine brain MRI in boys with CPP to 8 years (52). However, brain MRI should still be strongly considered in boys presenting with rapidly progressive puberty, the absence of a positive family history of CPP, or neurological signs and symptoms suggestive of CNS pathology. These recommendations require validation in larger, prospective, multicenter studies before they can be widely adopted in clinical practice.

## Emerging genes and polygenic models

Up to 25–30% of children with CPP exhibit familial aggregation, defined by at least one first- or second-degree relative with early pubertal onset (18). In boys, familial cases are particularly informative, as they are associated with a significantly lower probability of detecting intracranial lesions. Maternal age at menarche has repeatedly emerged as a surrogate marker of inherited pubertal timing.

The most robustly implicated genes in CPP encode inhibitors of pubertal initiation. Loss-of-function variants in *MKRN3* and *DLK1*—both paternally expressed imprinted genes—are now recognized as the most common monogenic causes of familial CPP in both sexes, with a notable prevalence in males (19, 20). Importantly, patients harboring pathogenic variants in these genes almost invariably show normal brain MRI findings, supporting the concept that genetic testing may, in selected cases, precede or even obviate neuroimaging.

Rare gain-of-function variants in *KISS1* and *KISS1R* have also been described, providing key mechanistic insights into kisspeptin-mediated activation of GnRH neurons (21).

Additional candidate genes involved in pubertal regulation include *LIN28B, GABRA1, NPY, TAC3, TACR3, PROKR2, and MAGEL2* (22). While pathogenic variants in these genes are rare, they highlight the complexity of the neuroendocrine network governing puberty. Increasing evidence suggests that CPP may represent the extreme phenotype of a polygenic continuum, in which the cumulative burden of common variants interacts with environmental exposures to precipitate early HPG axis reactivation.

In boys with idiopathic CPP—particularly those with early onset or strong familial clustering—targeted gene panels or exome sequencing can provide diagnostic clarification, guide genetic counseling, and support a precision medicine approach. Genetic testing may also help stratify the need for neuroimaging and long-term follow-up.

## Environmental and epigenetic modulators of pubertal timing

Endocrine-disrupting chemicals (EDCs) encompass a heterogeneous group of exogenous compounds that can perturb hormonal signaling at multiple levels of the hypothalamic–pituitary–gonadal (HPG) axis and have been increasingly investigated as potential modulators of pubertal timing (23). They can act as agonists or antagonists of estrogen receptors and may also interact with androgen and progesterone receptors. In addition, some EDCs can mimic endogenous estrogens and androgens or competitively inhibit the binding of endogenous hormones to their receptors, thereby exerting anti-estrogenic and anti-androgenic effects (24).

Through these mechanisms, EDCs can disrupt endocrine homeostasis and interfere with physiological pubertal development.

EDCs include plasticizers such as bisphenol A (BPA) and phthalates, pesticides, and other industrial chemicals that may exert estrogenic, anti-androgenic, or mixed endocrine effects (25–27).

Increasing epidemiological evidence supports an association between exposure to selected EDCs and altered pubertal timing, including precocious puberty. With specific regard to males, a nationwide cross-sectional study conducted in Korean adolescents reported that exposure to mixtures of BPA and phthalate metabolites was positively associated with precocious puberty in boys; however, effects estimates were imprecise and the cross-sectional design precluded causal conclusions (28).

In contrast, a large longitudinal cohort study from China demonstrated that persistent exposure to multiple phthalates was associated with an increased risk of early pubertal onset in boys, with evidence of synergistic interactions between phthalates and estradiol levels and antagonistic interactions with testosterone, suggesting disruption of normal androgen–estrogen balance during pubertal development (29).

A comprehensive systematic review and meta-analysis by Uldbjerg et al. evaluating prenatal and postnatal exposure to EDCs and pubertal timing in both sexes identified consistent associations between phthalate exposure and earlier puberty in boys, whereas evidence for other classes of EDCs was less robust (30). Importantly, the authors highlighted that evidence in boys remains limited and heterogeneous compared with girls, with relatively fewer longitudinal studies and substantial variability in exposure assessment and outcome definitions.

With regard to pesticides, epidemiological data and experimental models suggest that exposure to non-persistent pesticides, including pyrethroids, may accelerate pubertal onset in males by interfering with HPG axis signaling, although human data remain limited (31, 32).

Narrative and systematic reviews further highlight that diet represents a major source of exposure to phthalates and BPA, and that these compounds may contribute to earlier pubertal timing, particularly in the context of increasing childhood obesity, which represents a relevant confounding factor (33) (**Figure 2**).

Beyond epidemiological associations, experimental and translational studies support the biological plausibility of EDC involvement in pubertal regulation. EDCs have been shown to interfere with GnRH pulsatility, kisspeptin signaling, gonadotropin secretion, androgen receptor activity, aromatase function, and key steroidogenic enzymes, including steroidogenic acute regulatory protein (StAR) (34).

In addition, epigenetic modifications induced by early-life exposure to EDCs have been proposed as a mechanism linking environmental factors to long-term alterations in HPG axis activity, potentially influencing pubertal timing across critical developmental windows (35).

Taken together, current evidence supports a correlation between exposure to selected EDCs—most notably phthalates, BPA, PFAS and certain pesticides—and precocious puberty in boys (**Figure 2**).

Exposure to per- and polyfluoroalkyl substances (PFAS) has been increasingly investigated as a potential endocrine-disrupting factor influencing pubertal timing, although findings remain heterogeneous. Epidemiological studies suggest that PFAS exposure is more consistently associated with delayed pubertal onset, particularly in girls, possibly mediated by a reduction in estradiol and DHEAS levels (36). However, some data indicate a potential association with peripheral precocious puberty, especially in relation to specific PFAS mixtures and estrogenic activity, suggesting complex, non-monotonic effects (37).

Evidence in male populations is limited and less consistent: some cohort studies report no clear association, while others suggest delayed pubertal onset (e.g., reduced testosterone or later testicular development at ultrasounds) with higher PFAS burden (38).

Prenatal exposure studies indicate sex different effects and opposite-effects for each PFAS-substance in boys, with signals toward earlier pubertal onset for PFHxS and PFHpS and later pubertal onset for PFDA and PFNA exposure (39).

Most human evidence derives from biomonitoring studies measuring PFAS in serum (commonly PFOS, PFOA, PFHxS, PFNA), but no clear “critical threshold” has been established, and mixture effects appear relevant (40).

Experimental data are comparatively scarce but support biological plausibility: PFAS exhibit endocrine-disrupting properties, including interaction with steroidogenesis and estrogen receptors, and animal studies suggest effects on reproductive development and germline epigenetic regulation, although direct evidence on pubertal timing in rodents remains limited (41).

Nonetheless, substantial gaps persist regarding exposure assessment, dose–response relationships, sex-specific effects, and the distinction between central precocious puberty and other forms of early pubertal development, underscoring the need for well-designed longitudinal studies with standardized clinical endpoints.

## Clinical implications: toward a personalized diagnostic algorithm

Integrating epidemiological, genetic, and environmental data supports a personalized approach to the evaluation of boys with CPP. Brain MRI should be strongly considered in boys with onset before 8 years, rapid pubertal progression, neurological symptoms, or absence of familial predisposition. In contrast, boys presenting after 8 years of age with slow progression and positive family history may benefit from prioritized genetic evaluation and careful clinical monitoring.

Such a risk-based algorithm aligns with principles of precision medicine and has the potential to optimize diagnostic efficiency while minimizing unnecessary investigations (**Figure 1**).

## Future directions and unmet needs

Key priorities for future research include longitudinal studies assessing environmental exposures in boys, large-scale genetic studies to refine genotype–phenotype correlations, and the integration of polygenic risk scores into clinical practice. Updated international consensus guidelines are urgently needed to reflect contemporary evidence and harmonize the management of male CPP.

## Conclusions

CPP in boys is increasingly recognized as a multifactorial condition driven by the interplay of genetic predisposition and environmental modulation. Contemporary evidence demonstrates that the prevalence of intracranial lesions is substantially lower than historically reported, supporting a selective, risk-based approach to neuroimaging. Advances in genetics have identified key molecular regulators of pubertal timing, while emerging data suggest that environmental and early-life factors may further influence HPG axis activation. Integrating these insights into clinical practice is essential to promote personalized, evidence-based management of boys with CPP.
