# Supplementary material for: Precocious puberty in boys: current insights into etiology, genetic advances, and environmental factors
Source: Front Endocrinol (Lausanne). 2026 Jul 16;17:1874027. doi: 10.3389/fendo.2026.1874027 (PMC13422188; doi:10.3389/fendo.2026.1874027)
Supplement: Supplementary file 1 [file Table1.docx]

**Supplementary Table 1:** the percentage of CPP stratified for etiology in male and female population.

| **Brain MRI findings** | **Percentage of Precocious Puberty Cases in females (2)** | **Percentage of Precocious Puberty Cases in males reported in literature before 2020 (3)** | **Percentage of Precocious Puberty Cases in males in the most recent studies in Italy (5,13)** |
| --- | --- | --- | --- |
| **Not relevant alterations** | 80-90% | 30-50% | 74.6- 86% |
| **Mild alterations** | 10-20% | 33-85% | 8- 17.6% |
| **Brain pathological lesions and tumors** | 5-10% | 50-70% | 5.7- 7.8% |
